# Complete genomes of two Ikeda-genotype *Orientia tsutsugamushi* isolates from South Korea reveal within-lineage divergence and contrast with the Boryong reference strain

**DOI:** 10.1371/journal.pone.0351070

**Published:** 2026-07-09

**Authors:** Hyungsuk Kang, Yeon-Joo Choi, Kyeonghye Guk, Misoon Kim, Kwangjun Lee, Won-Jong Jang

**Affiliations:** 1 Department of Microbiology, School of Medicine, Konkuk University, Chungju, Republic of Korea; 2 Research Institute of Medical Science, School of Medicine, Konkuk University, Chungju, Republic of Korea; 3 Division of Zoonotic and Vector Borne Disease Research, National Institute of Health, Korea Disease Control and Prevention Agency, Osong, Cheongju, Republic of Korea; 4 Division of Research and Development, LabOntier, Seoul, Republic of Korea; Huadong Research Institute for Medicine and Biotechniques, CHINA

## Abstract

*Orientia tsutsugamushi* strain Ikeda is a scrub typhus reference strain originally described in Japan, and Ikeda *56-kDa* type-specific antigen sequence types have also been reported in South Korea. However, complete genome resources for South Korean Ikeda-genotype isolates remain limited. Here, we generated complete genomes for two archived clinical *O. tsutsugamushi* isolates from northern South Korea, CH219 and K4-135, using a PacBio HiFi and Illumina hybrid assembly approach and compared them with the Japanese reference strain Ikeda and the South Korean reference strain Boryong. Both genomes were assembled as single circular chromosomes and contained a substantial fraction of duplicated identical coding sequences, consistent with the highly repetitive nature of *O. tsutsugamushi* genomes. Strains CH219 and K4-135 had identical *56-kDa* TSA sequences and MLST profiles to those of the Ikeda reference strain and clustered within the Ikeda-associated clade in recombination-filtered core-genome phylogeny and ANI analyses. Within this comparison, the two South Korean isolates showed more closely related to each other than to the Japanese Ikeda reference genome. Whole-genome dot plots further indicated structural variation among the Ikeda-associated genomes. Insertion sequence (IS) profiling showed differences in IS-related CDS copy number patterns between the Boryong reference genome and the Ikeda-associated genomes, with strain Boryong showing a higher observed number of ISOt6-related CDS hits and fewer high-identity ISOt3-related hits under the applied thresholds. Together, these genomes provide new resources for Ikeda-like *O. tsutsugamushi* strains detected in South Korea and support the need for expanded complete and well-supported whole-genome data to better understand genome diversity in scrub typhus agents.

## Introduction

Scrub typhus is a mite-borne zoonotic disease endemic across the Asia-Pacific region, including South Korea [1]. Clinically, it often presents with non-specific symptoms such as acute fever, myalgia, and headache, and can be difficult to distinguish from other acute febrile illness co-circulating in endemic settings, except when an eschar is present [2,3].

The etiologic agent, *Orientia tsutsugamushi*, is an obligate intracellular bacterium [4] and displays substantial genetic diversity. For strain-level classification and molecular epidemiology, genotyping based on the *56-kDa* type-specific antigen (TSA) gene has been widely used [5–8]. Several studies have suggested genotype- or strain-associated differences may influence in virulence and clinical manifestations, for example, the Boryong strain has been linked to more severe clinical outcomes than the Karp strain in some reports [9], and the Kato and Ikeda strains have been described as relatively virulent compared with the avirulent TA686 strain [10].

In South Korea, the Boryong strain is frequently reported as a predominant genotype, while minor genotypes such as Karp, Gilliam, Je-cheon, and Young-worl have also been detected in human patients [7,11–13]. The Ikeda strain, originally described in Japan, was first reported in South Korea in 2012 from a severe scrub typhus case [14], and experimental studies in Japan have suggested high virulence of the Ikeda strain in mouse models [15,16]. However, genome resources for strain Ikeda clinical isolates from South Korea remain limited.

At the genomic level, *O. tsutsugamushi* possesses a large and unusually repetitive chromosome with extensive rearrangements [16–18]. Comparative analyses of the Boryong and Ikeda strains have shown that, despite major structural variation, these strains share a conserved core gene set alongside large repetitive sequences, including duplicated genes and *O. tsutsugamushi* amplified genetic elements (OtAGE) [16–18]. Much of this repetitiveness is driven by mobile genetic elements such as insertion sequences (ISs) [16,18], which can contribute to genome expansion, rearrangements, and pseudogene formation [17,19]. IS insertions may also disrupt coding sequences or influence the expression of neighboring genes involved in antigenicity and host-pathogen interactions [20]. Because these genome-scale features are not captured by single-locus typing, whole genome sequencing (WGS) provides a complementary framework to resolve strain relatedness and to characterize repetitive and mobile-element landscapes.

Here, we generated complete genome sequences of two archived clinical Ikeda-like *O. tsutsugamushi* isolates (CH219 and K4-135), originally obtained from scrub typhus patients in South Korea, using a long- and short-read hybrid assembly approach (PacBio HiFi and Illumina). We aimed to define their genomic relatedness to the Japanese reference strain Ikeda (NC_010793.1) and to the predominant South Korean strain Boryong (NC_009488.1), focusing on (i) core-genome similarity and phylogenetic placement, (ii) genome structure and repetitive gene content, and (iii) IS family composition and copy-number patterns. By providing complete genome resources for Ikeda-like strains detected in South Korea, this study supports comparative analyses of *O. tsutsugamushi* diversity, genome evolution, and genomic surveillance.

## Methods

### Bacterial isolates, cell culture, and genomic DNA extraction

Two *O. tsutsugamushi* isolates were included in this study and designated *O. tsutsugamushi* strain CH219 and strain K4-135. These isolates were archived, de-identified clinical isolates originally obtained from scrub typhus cases in Wonju (Gangwon Province, 2023) and Goyang (Gyeonggi Province, 2024), respectively, during a previous study in South Korea, and stored as frozen stocks at a collaborating institution. Because these materials were archival and de-identified, we did not have access to identifiable patient records or travel history information. For this genomic study, no new human specimens were collected, and no identifiable patient information was accessed.

Frozen stocks were thawed and inoculated onto Vero cell monolayers, followed by incubation at 34°C with 5% CO_2_. Infection in Vero cell culture was monitored microscopically for cytopathic effect (CPE). Propagation of the isolates was suspected when CPE was observed and was confirmed by PCR amplification of the *56-kDa* TSA gene using primers WJ173F (5’–CCAGGATTTAGAGCAGAG–3’) and WJ794R (5’-CTAGAAGTTATAGCGTACACCTGCACTTGC-3’), as previously described [21], targeting an approximately 1,200 bp region of the *56-kDa* TSA gene. PCR cycling conditions were as follows, initial denaturation at 94°C for 5 minutes, 40 cycles of 94°C for 30 seconds, 52°C for 30 seconds, and 72°C for 90 seconds, followed by a final extension with 72°C for 3 minutes. The expected amplicons were visualized on agarose gel electrophoresis. The PCR product was further confirmed by Sanger sequencing and BLASTn analysis against the NCBI GenBank nr/nt database [22].

Genomic DNA was extracted from infected cell culture material using the DNeasy Blood and Tissue Kit (QIAGEN, Hilden, Germany), according to the manufacturer’s instructions. The extracted DNA was submitted to Macrogen Inc. (Seoul, South Korea) for sequencing. DNA quality control at Macrogen was performed prior to sequencing, including concentration measurement using a Qubit fluorometer and fragment-size assessment by pulsed-field gel electrophoresis. No additional purification step was performed prior to sequencing.

### Whole genome sequencing, assembly, and annotation

Whole-genome sequencing was performed by a commercial provider Macrogen using PacBio HiFi long-read sequencing on the PacBio Revio platform (Pacific Biosciences, CA, USA) and Illumina paired-end sequencing (2 x 150 bp) on the NovaSeq X Series platform (Illumina, CA, USA). Libraries were prepared using the SMRTbell Prep Kit 3.0 (PacBio) and the TruSeq DNA Nano Kit (Illumina) according to the manufacturer’s protocols. According to the provider reports, routine preprocessing included quality filtering and adapter trimming, however, no separate host-read depletion step against the Vero cell genome was explicitly described.

Assembly and polishing were performed by the sequencing provider. According to the provider, multiple assembly and polishing workflows were evaluated for each isolate, and the final assemblies with the best assembly statistics, including contiguity, circularization status, read-mapping coverage, and overall quality metrics, were selected for downstream analysis. For strain K4-135, PacBio HiFi reads were assembled de novo using Flye v2.9 [23]. The initial assembly improved by read mapping and polishing with Racon (version not provided by the sequencing provider), followed by additional polishing with Inspector v1.0.1 [24] and Pilon v1.22 [25]. For strain CH219, PacBio HiFi reads were assembled using the Microbial Genome Analysis application in SMRT Link v25.1.0.257715, followed by error correction with Inspector v1.0.1 and additional polishing with Pilon v1.22. Illumina reads were quality-checked with FastQC v0.11.7 [26] and trimmed using Trimmomatic v0.38 [27] before short-read polishing.

The final assemblies were evaluated using read self-mapping, BLAST v2.14.0 [22], ANI analysis with pyani v0.2.7 [28], and BUSCO v5.1.3 [29], as described in the provider reports. Both assemblies were recovered as a single circularized contig with 100% read-mapping coverage in the validation summary.

All downstream analyses starting from the final assemblies were performed in-house, primarily using the Galaxy platform [30]. Gene prediction and structural annotation of the final assemblies were carried out with Prokka v1.14.6 [31]. Functional annotation of predicted coding sequences was performed using InterProScan v5.34-73.0 [32] and EggNOG v4.5 [33] to assign protein domains and orthologous groups. Prokka was used to provide a consistent annotation framework across all genomes included in the comparative analyses and to generate CDS-level output files required for downstream clustering and functional annotation workflows. Because *O. tsutsugamushi* has a highly repetitive genome with numerous potentially degraded or pseudogized loci, this annotation strategy was used primarily for standardized comparative analysis rather than definitive pseudogene annotation. PGAP annotation outputs available through the NCBI submission workflow were reviewed as an additional reference for structural annotation.

### Identification and quantification of identical duplicated CDSs

Gene-level redundancy was assessed using the Prokka-generated nucleotide sequences of coding sequences (CDSs; ‘.ffn’ files) from each genome. CDS sequences were clustered with CD-HIT-EST v4.8.1 [34] at 100% sequence identity (-c 1.0), with all other parameters left at their default settings, such that each cluster represented identical CDS copies within a genome. Clusters with at least 2 members were considered duplicated CDS clusters, and cluster size was used as a proxy for copy number. CD-HIT cluster output files (‘.clstr’) were parsed to summarize cluster sizes, list member locus tags, and identify high-copy clusters.

To assign functional information to duplicated CDS clusters, CD-HIT cluster membership tables were merged with genome annotation tables using locus tags as keys, linking each CDS to its Prokka and EggNOG annotations (e.g., locus_tag, gene, product, and EggNOG functional description). This produced a non-redundant catalog of duplicated CDS clusters with associated functional annotations for comparative analyses among the CH219, K4-135, and the reference genomes Boryong and Ikeda strains. For summary statistics, the number of duplicated genes was defined as the total number of CDSs belonging to CD-HIT clusters with at least 2 members, whereas the number of duplicated clusters represented the number of such clusters. In addition, the summed nucleotide length of CDSs belonging to duplicated clusters was calculated for each genome and expressed as a proportion of both total genome length and total annotated CDS length.

Genes were classified as hypothetical if the Prokka ‘product’ field was ‘hypothetical protein’ and EggNOG provided no informative functional assignment (i.e., empty, ‘-’, or ‘uncharacterized’). Genes with a specific functional assignment in at least one annotation source were classified as functionally annotated.

### Phylogenetic and basic comparative analyses

Phylogenetic comparisons of strains CH219 and K4-135 with publicly available *O. tsutsugamushi* genomes were performed at three levels, (i) a single-locus analysis based on the *56-kDa* TSA gene, (ii) a multilocus analysis using the seven-gene *O. tsutsugamushi* sequence typing (MLST) scheme (*gpsA*, *mdh*, *nrdF*, *nuoF*, *ppdK*, *sucB*, and *sucD*), and (iii) a whole-genome framework based on a concatenated core-gene alignment with recombination filtering.

Public genomes were identified by querying the NCBI Assembly database [35] (accessed on December 12, 2025), yielding 30 assemblies assigned to *Orientia tsutsugamushi*. Assemblies were screened for assembly status, and only those labeled as ‘Complete Genome’ or ‘Chromosome’ were retained for further consideration (n = 17). Assemblies labeled as scaffold-, contig-, or draft-level were excluded because they were not suitable for chromosome-level comparative analysis. Among the 17 retained assemblies, duplicate submissions representing the same strain were further reviewed based on strain designation and assembly metadata, and one redundant entry was removed. This resulted in 16 non-redundant reference genomes for downstream analyses (**S1 Table**).

For the *56-kDa* TSA gene and the seven MLST loci, gene sequences were extracted from the annotated genome assemblies by manually identifying the corresponding loci in the Prokka annotation outputs, with additional confirmation based on EggNOG functional annotation. The extracted loci were cross-checked against the *O. tsutsugamushi* NCBI and PubMLST databases to confirm locus identity and allele assignment. Reference gene sequences from the 16 publicly available genomes were retrieved using the same annotation-based approach.

For the *56-kDa* TSA gene phylogeny, representative and geographically relevant *56-kDa* TSA sequences were selected from public nucleotide repositories, with emphasis on strains from South Korea, Japan, and nearby regions, including sequences closely related to the Ikeda strain. In addition, *56-kDa* TSA sequences were retrieved from the 16 reference genome assemblies included in this study. A total of 31 *56-kDa* TSA gene sequences were aligned using MUSCLE in MEGA12 [36], and a maximum-likelihood tree was inferred under the GTR + G + I model selected by MEGA12, with 1,000 nonparametric bootstrap replicates.

For MLST analyses, the seven housekeeping loci were not newly amplified or sequenced using MLST-specific primers, instead, all allele sequences were retrieved *in silico* from the annotated whole-genome assemblies. Locus identity and allele assignment were then cross-checked against the *O. tsutsugamushi* PubMLST database [37]. For multilocus sequence analysis (MLSA), the seven housekeeping loci were concatenated, aligned using MUSCLE in MEGA12, and analyzed by maximum-likelihood inference under T92 + G + I model selected by MEGA12, with 1,000 nonparametric bootstrap replicates.

For whole-genome phylogeny and recombination-aware comparisons, Prokka generated ‘.gff’ files for all 18 genomes (CH219, K4-135, and 16 reference genomes) were processed with Roary v3.13.0 [38] to identify core genes and generate a concatenated core-gene nucleotide alignment (minimum BLASTP identity 95%; core genes defined as present in 100% of genomes). To compare phylogenies before and after recombination filtering, a maximum-likelihood tree was first reconstructed from the Roary core-gene alignment (before recombination filtering) using IQ-TREE v2.4.0 [39] on the Galaxy platform with ModelFinder [40] to select the best-fit model; the selected model for this alignment was GTR + F + I + R7, and branch support assessed using 1,000 nonparametric bootstrap replicates. Recombination was then detected and filtered using Gubbins v3.2.1 [41] with default settings; 5 iterations, Weighted Robinson-Foulds convergence criterion, RAxML as the tree builder, a minimum of three SNPs, filter percentage of 25, minimum and maximum window sizes of 100 and 10,000 bp, *p-value* threshold of 0.05, trimming ratio of 1.0, no extensive search, and no removal of identical sequences. The resulting recombination-filtered polymorphic-sites alignment was used to reconstruct the ‘after recombination filtering’ phylogeny in IQ-TREE under the best-fit model selected by ModelFinder; for this alignment, the selected model was TVM + F + ASC + R3, and branch support was assessed using 1,000 nonparametric bootstrap replicates. Because the recombination-filtered alignment contained only variable sites, ascertainment bias correction was applied as implemented in IQ-TREE (ASC). The final tree was visualized and edited in MEGA12.

### Average nucleotide identity (ANI)

Pairwise ANI values were calculated among the 18 WGS datasets (16 reference genomes plus CH219 and K4-135 from this study). ANI values were computed using FastANI v1.3 [42] on the Galaxy platform with default parameters. The resulting ANI matrix was imported into RStudio v2025.09.2 Build 418 [43] and visualized as a heatmap using ggplot2 v3.5.2 [44], with each cell colored according to the ANI value (white-to-blue gradient, 98.5–100%) and overlaid with the corresponding ANI percentage.

### Pairwise Single nucleotide polymorphism (SNP) distance

Pairwise SNP distances were calculated from the recombination-filtered polymorphic-sites alignment generated by Gubbins. The number of SNP differences between each pair of genomes was computed as the count of mismatched nucleotide sites in the alignment using Galaxy SNP distance matrix v0.8.2 [45] with default settings. For concise presentation, SNP distances between each reference genome and the two South Korean Ikeda-like isolates (CH219 and K4-135) were summarized, while the complete pairwise matrix for all genomes is provided in Supplementary **S2 Table**. Values represent the number of nucleotide differences between genome pairs in the recombination-filtered variable-sites alignment.

### Whole-genome alignment and dot plot visualization

Pairwise whole-genome alignments were generated on a Linux environment (Ubuntu v24.04.1 LTS) using MUMmer v3.23 [46] (nucmer v3.1, delta-filter, show-coords, and mummerplot v3.5 modules). Prior to alignment, FASTA headers were standardized and, when necessary, circular chromosomes were rotated to a consistent start position near the predicted replication origin region (*dnaA*/putative *ori*) using Circlator v1.5.6-11 [47]. Genome pairs were aligned with nucmer (--mum) using a minimum match length of 100 bp (-l 100) and cluster size of 500 bp (-c 500). Alignments were filtered with delta-filter (−1) using a minimum alignment length of 2,000 bp and a minimum nucleotide identity of 95%. Filtered alignment coordinates were examined using show-coords (-rcl), and dot plots were generated with mummerplot v3.5 using the --png, --layout, and --filter options. Dot plots were interpreted qualitatively as indicators of structural differences in genome organization rather than as formal breakpoint-mapping analyses.

### Identification and quantification of IS-related CDSs

Predicted CDSs were obtained from Prokka annotations as nucleotide FASTA files (‘.ffn’) and were queried against the ISfinder web interface [48] (accessed on November 26^th^, 2025) using the BLASTn program against the ISfinder database. The BLASTn setting were left at the web-interface defaults, except that the maximum E-value threshold was changed to 1 x 10^-4^ (alignment view: pairwise; word size: 11; gap costs; existence 5, extension 2; query filtering disabled). For each CDS, only the top-scoring ISfinder hit was retained. In the primary analysis, CDSs were classified as IS-related (transposase-like CDSs) if the top hit met all criteria, E-value < 1x10^-4^, alignment length ≥ 300 bp, and nucleotide identity ≥ 90%. To assess robustness of IS-family assignments under higher stringency, the same results were further filtered using two additional criteria; (i) alignment length ≥ 300 bp and nucleotide identity ≥ 99%; and (ii) alignment length ≥ 400 bp and nucleotide identity ≥ 99%. IS-related CDSs were assigned to IS families according to the annotation of the retained top hit, and per-genome copy numbers for each IS family were summarized using custom scripts in Rstudio environment [43]. IS family profiles were compared among strains CH219 and K4-135, and the reference genomes Ikeda and Boryong.

## Results

### General genomic features of *O. tsutsugamushi* strains CH219 and K4-135

PCR amplification of the *56-kD*a TSA gene yielded partial products of 1,221 bp for strain K4-135 (GenBank accession: PZ294055) and 1,276 bp for strain CH219 (accession: PZ294054). Comparison of these PCR-derived sequences with the corresponding WGS-derived *56-kDa* TSA sequences (both 1,551 bp) showed complete identity across the overlapping regions in both isolates. Thus, although the PCR products did not span the full target region, no sequence discrepancy was observed between the PCR-derived and WGS-derived sequences within the recovered regions. The complete *56-kDa* TSA CDSs used in the comparative analyses were derived from WGS assemblies, whereas the PCR-derived sequences were used only for preliminary confirmation.

Both *O. tsutsugamushi* strains CH219 and K4-135 were assembled as single, circular chromosomes (**S1 Fig**). According to the provider’s hybrid assembly QC reports, both assemblies achieved 100% genome coverage by both Illumina and PacBio reads (**S3 Table**). For strain CH219, Illumina sequencing generated 19,175,094 paired-end read pairs, 5,355,442 reads (27.9%) were mapped to the final assembly according to the provider’s mapping summary, corresponding to 100% breadth of coverage and a mean depth of 393.9x. PacBio HiFi sequencing generated 110,128 reads, with 35,395 reads (32.1%) mapped and a mean depth of 169.7x. For strain K4-135, Illumina sequencing generated 16,957,842 read pairs, with 6,899,490 reads (40.7%) mapped (100% breadth; 475.6x mean depth), while PacBio HiFi sequencing generated 76,438 reads, with 34,508 mapped reads (45.2%) mapped (100% breadth; 164.9x mean depth) (S3 Table). These mapping statistics were taken directly from the provider’s self-mapping summaries. Although the provider reports describe quality filtering and adapter trimming, they do not specify a separate host-read depletion step prior to assembly. Therefore, the relatively low mapped fractions likely reflect the presence of non-target background reads, including residual host-cell DNA from Vero cell culture preparations. At the same time, the complete breadth of coverage and high mean depth indicate that the final chromosome assemblies were well supported by the sequencing data.

The genome of strain CH219 was 1,978,415 bp in length, slightly smaller than that of strain K4-135 (2,059,857 bp), with GC contents of 30.5% and 30.6%, respectively (**Table 1**). In the context of publicly available genomes, the chromosome lengths of strains CH219 and K4-135 fell within the reported range for *O. tsutsugamushi*, 1,932,116 bp in strain UT176 (accession no. NZ_LS398547.1) to 2,469,803 bp in strain Karp (NZ_LS398548.1) (S1 Table). The Japanese reference strain Ikeda (2,008,987 bp; NC_010793.1) was comparable in size to the two South Korean strains, whereas the South Korean reference strain Boryong (2,127,051 bp; NC_009488.1) was larger.

**Table 1:** Basic genomic information of *O. tsutsugamushi* strains CH219 and K4-135 from this study and the reference strains Ikeda and Boryong.

	strain CH219	strain K4-135	strain Ikeda (NC_010793.1)	strain Boryong (NC_009488.1)
Source	Human, South Korea	Human, South Korea	Human, Japan (18)	Human, South Korea (17)
First WGS data reported	2025 (current study)	2025 (current study)	2008 (18)	2007 (17)
Length (bp)	1,978,415	2,059,857	2,008,987	2,127,051
GC ratio (%)	30.5	30.6	30.5	30.5
Total annotated genes	2,198	2,260	2,222	2,479
tRNA	34	34	34	34
rRNA	2	2	2	2
CDSs	2,162	2,224	2,186	2,443
Functionally assigned	1,810	1,857	1,823	1,952
Hypothetical^*^	352	367	363	491
Repeated genes^**^	538 (159 clusters)	520 (171 clusters)	501 (163 clusters)	882 (238 clusters)
Functionally assigned	451 (131 clusters)	433 (145 clusters)	444 (141 clusters)	676 (184 clusters)
Hypothetical^*^	87 (28 clusters)	87 (26 clusters)	66 (22 clusters)	206 (54 clusters)
Singleton genes	1,660	1,740	1,721	1,597
Functionally assigned	1,398	1,460	1,425	1,312
Hypothetical^*^	262	280	296	285
Annotated CDS total length (bp)^***^	1,516,526	1,590,260	1,544,852	1,614,201
Total length of repeated CDSs (bp)^***^	311,631	297,774	306,723	461,814
Repeated CDS length/ genome size (%)	15.8%	14.5%	15.3%	21.7%
Repeated CDS length/ total annotated CDS length (%)	20.5%	18.7%	19.9%	28.6%

* Hypothetical genes were defined as CDSs annotated as ‘hypothetical protein’ by Prokka and lacking any specific functional assignment in EggNOG (empty, ‘-’ or ‘Uncharacterized’).

** Repeated genes were defined as coding sequences belonging to CD-HIT-EST clusters of sequences sharing 100% nucleotide identity (-c 1.0), with at least two members.

*** Repeated CDS length was calculated as the summed nucleotide length of CDSs belonging to CD-HIT-EST clusters with at least two members at 100% nucleotide identity.

EggNOG-based functional categorization showed broadly similar profiles between strain CH219 and strain K4-135 (**S2 Fig**). In both genomes, the most represented categories were S (functional unknown) and L (replication, recombination and repair), followed by T (signal transduction mechanisms) and U (intracellular trafficking, secretion, and vesicular transport). These results indicate that the two Ikeda-related genomes share a highly similar overall functional repertoire despite differences in genome size and structural organization.

Prokka annotation identified 2,198 and 2,260 total annotated genes in strains CH219 and K4-135, including 34 tRNA and 2 rRNA genes in each genome. The number of CDSs was 2,162 for CH219 and 2,224 for K4-135, respectively. Across the four strains (CH219, K4-135, Ikeda, and Boryong), the total number of annotated genes broadly tracked genome size (Table 1). Notably, the proportion of duplicated identical CDSs differed among strains. Strains Ikeda, CH219 and K4-135 showed lower fractions of duplicated CDSs than strain Boryong in this comparison, indicating a greater repeated CDS burden in the Boryong reference genome.

CDS-level redundancy analysis further showed that repeated CDSs accounted for 311,631 bp (15.8% of the genome, 20.5% of the total annotated CDS length) in strain CH219 and 297,774 bp (14.5% of the genome, 18.7% of the total annotated CDS length) in strain K4-135 (Table 1). The corresponding repeated CDS lengths were 306,723 bp (15.3% of the genome, 19.9% of the total annotated CDS length) in strain Ikeda and 461,814 bp (21.7% of the genome, 28.6% of the total annotated CDS length) in strain Boryong. These results indicate substantial CDS-level repetition across all four genomes, with strain Boryong showing the largest repeated CDS content among the four strains compared.

### Phylogenetic analysis

**Fig 1:**
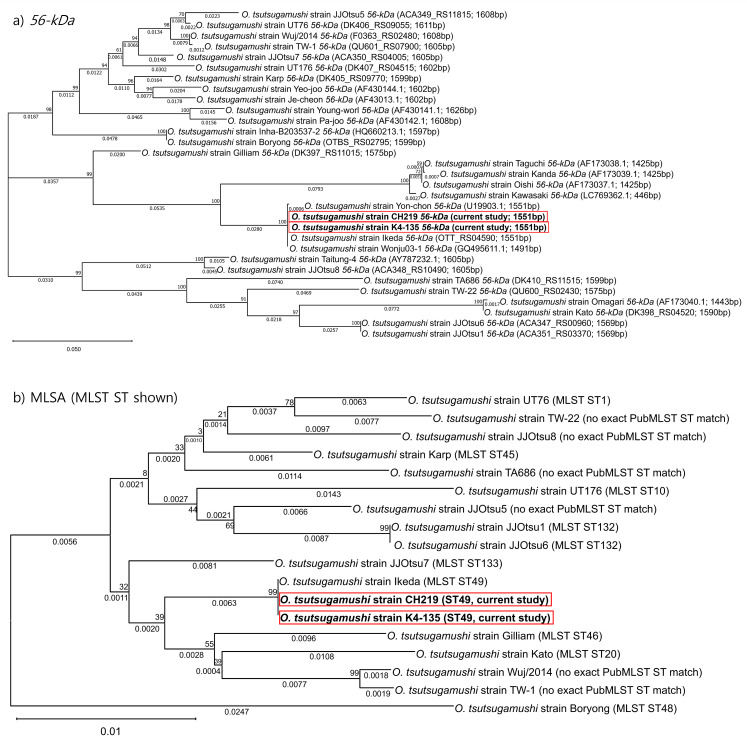
Single-locus and multilocus phylogenetic placement of the South Korean Ikeda-genotype *Orientia tsutsugamushi* isolates. Maximum-likelihood trees were constructed using (a) the *56-kDa* TSA gene (single gene), (b) multilocus sequence analysis (MLSA) based on concatenated sequences of the seven MLST *loci* (*gpsA*, *mdh*, *nrdF*, *nuoF*, *ppdK*, *sucB*, and *sucD*). For the study isolates, *56-kDa* TSA and MLST locus sequences were extracted from the whole-genome assemblies, and MLST alleles and sequence types were assigned in silico using the *O. tsutsugamushi* PubMLST database. For some reference genomes, no exact PubMLST sequence type match was available based on the *in silico* allele profile, theses strains are labeled as ‘no exact PubMLST ST match’. The two South Korean Ikeda-genotype strains (CH219 and K4-135) are highlighted.

**Fig 2:**
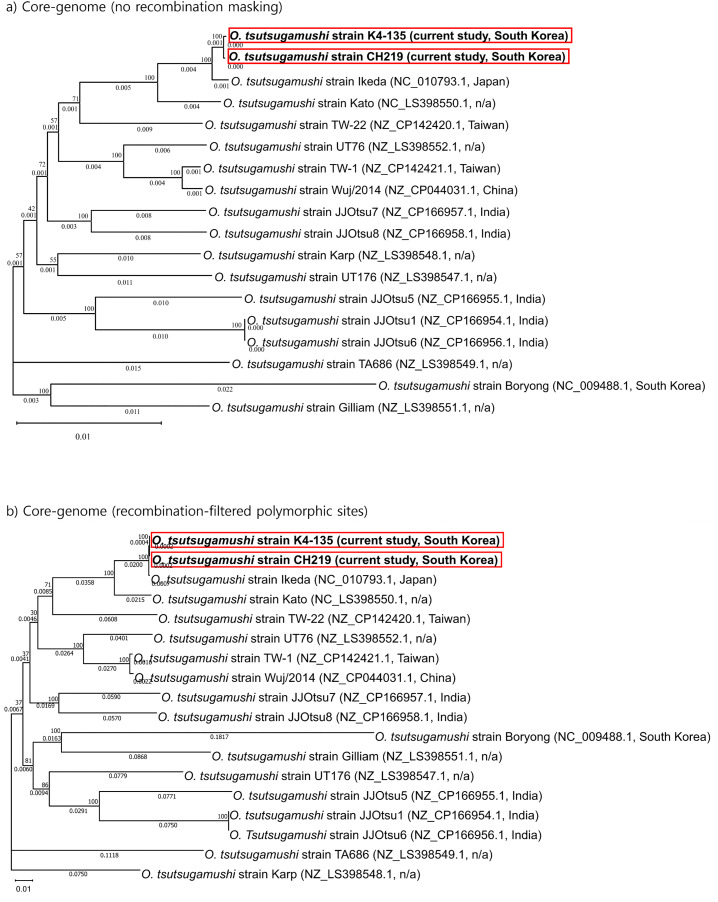
Core-genome phylogeny before and after recombination filtering. Maximum-likelihood phylogenies were inferred from (a) the Roary concatenated core-gene nucleotide alignment (540 core genes, pre-filtering) and (b) the Gubbins recombination-filtered polymorphic-sites alignment derived from the same core gene alignment (post-filtering). Node labels indicate standard bootstrap support (%) from 1,000 replicates. The two South Korean Ikeda-genotype strains (CH219 and K4-135) are highlighted.

The phylogenetic positions of the two South Korean strains CH219 and K4-135 were assessed at three levels, a single-locus tree based on the *56-kDa* TSA gene, a seven-locus MLSA using the *O. tsutsugamushi* MLST scheme (**Fig 1**), and a whole-genome phylogeny based on the concatenated core-gene (540 genes) alignment before and after recombination filtering polymorphisms across 16 publicly available genomes and the two strains from this study (**Fig 2**).

In the *56-kDa* TSA gene tree (Fig 1a), strains CH219 and K4-135 shared an identical WGS-derived *56-kDa* TSA sequence with the Japanese reference strain Ikeda (OTT_RS04590; 1,551 bp) and clustered with Japanese strains including Taguchi, Kanda, Oishi, and Kawasaki. In contrast, the major South Korean reference strain Boryong clustered with other South Korean *56-kDa* TSA gene sequences, including Young-worl, Je-cheon, Yeo-joo, together with the Karp strain that has also been detected in South Korea.

At the MLST level (Fig 1b), strains CH219 and K4-135 again showed an identical allelic profile to strain Ikeda and were assigned to sequence type ST49, whereas strain Boryong was assigned to ST48. In the MLSA phylogeny inferred from concatenated sequences of the seven loci (Fig 1b), strains CH219 and K4-135 grouped tightly with strain Ikeda, while strain Boryong formed a distinct branch, indicating separation from the Ikeda-associated genomes included in this analysis.

In the core-genome phylogeny inferred from the Roary core-gene alignment without recombination masking (Fig 2a), strains CH219 and K4-135 grouped with the Japanese reference strain Ikeda (NC_010793.1) and strain Kato (NC_LS398550.1). After recombination filtering with Gubbins (Fig 2b), the overall placement of strains CH219 and K4-135 within the Ikeda-associated cluster was maintained, although branch lengths and support values changed for several internal nodes following removal of recombination-associated polymorphism. Notably, strains CH219 and K4-135 each formed short but distinct branches within this clade in both analyses, consistent with minor genome-wide divergence relative to strain Ikeda.

This pattern was supported by pairwise SNP distances, with strains CH219 and K4-135 differing by 22 SNPs and differing from Ikeda by 78 and 76 SNPs, respectively (S2 Table and **Table 2**). In contrast, strain Boryong (NC_009488.1) formed a substantially longer branch and grouped closer to strain Gilliam (NZ_LS398551.1), consistent with greater genome-wide divergence between Boryong and the Ikeda-associated cluster.

**Table 2:** Pairwise SNP differences between *O. tsutsugamushi* strains CH219 and K4-135 and other 16 reference genomes calculated from the Gubbins recombination-filtered polymorphic-sites alignment.

Strains	CH219	K4-135
CH219	–	22
K4-135	22	–
Ikeda	78	76
Kato	2,429	2,394
TW-1	4,976	4,951
Wuj_2014	4,978	4,949
TW-22	5,751	5,707
JJOtsu7	6,004	5,980
UT76	6,084	6,035
JJOtsu8	6,259	6,200
Karp	6,585	6,508
UT176	7,070	7,004
Gilliam	7,201	7,183
JJOtsu5	7,778	7,737
JJOtsu1	7,824	7,745
JJOtsu6	7,829	7,750
TA686	8,076	7,971
Boryong	10,598	10,491

### Average nucleotide identity among the 18 *O. tsutsugamushi* strains

**Fig 3:**
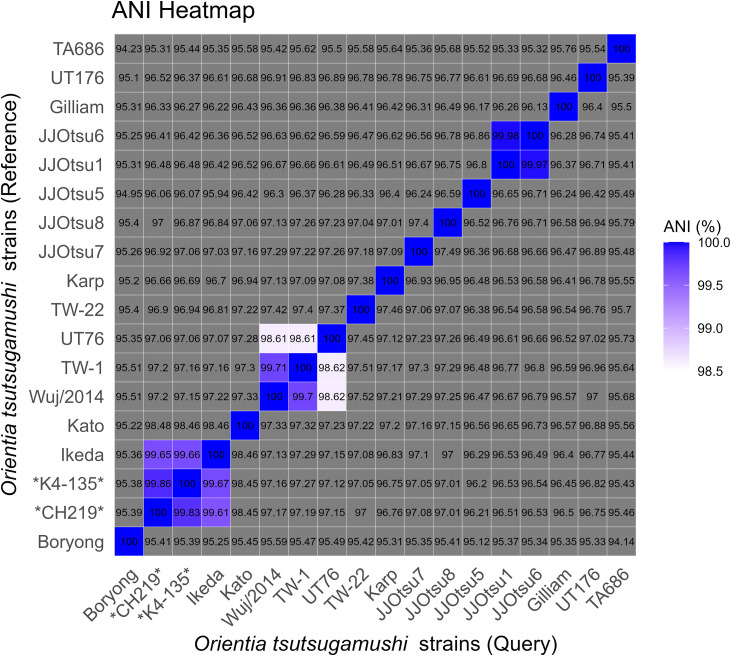
Pairwise Average Nucleotide Identity (ANI) heatmap for 18 *Orientia tsutsugamushi* strains, including the two South Korean strains from this study (CH219 and K4-135) and 16 publicly available genomes. ANI was calculated using FastANI, and higher nucleotide identity values are represented by darker blue colour. The color scale indicates ANI values among the genomes, with grey shading representing ANI values below 98.5%. The two South Korean strains CH219 and K4-135 are highlighted with *.

The pairwise ANI heatmap (**Fig 3**) showed a pattern consistent with the recombination-filtered core-genome phylogeny (Fig 2b) and pairwise SNP comparison. The CH219 and K4-135 strains exhibited the highest ANI values to each other (99.8–99.9%) and showed very high nucleotide identity to the Japanese reference strain Ikeda (99.6–99.7%). Both strains were also closely related to strain Kato, with ANI values around 98.5%. In contrast, the South Korean reference strain Boryong showed substantially lower ANI values to the Ikeda/Kato-associated clade (typically less than 96% across comparisons) than did strains CH219, K4-135, and Ikeda to each other, consistent with its greater genome-wide divergence observed in the SNP distance analysis (Table 2).

### Pairwise whole-genome dot plot comparison among four *O. tsutsugamushi* strains

**Fig 4:**
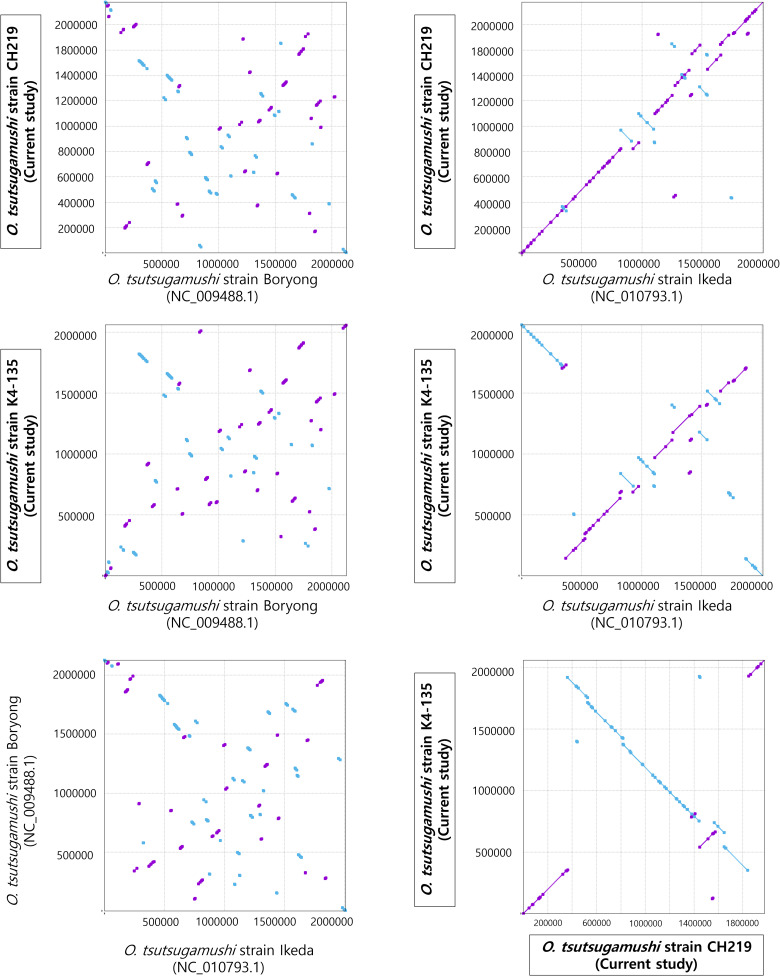
Pairwise whole-genome dot plots of four *Orientia tsutsugamushi* genomes. Dot plots were generated using MUMmer (nucmer) pairwise alignments filtered to retain matches with ≥ 95% nucleotide identity and ≥ 2 kb alignment length. Each panel shows genome-wide alignments between the indicated genome pair; forward matches are shown as purple and reverse-oriented matches in blue. The two South Korean Ikeda-genotype strains (CH219 and K4-135) are highlighted in bold.

Pairwise whole-genome dot plots were generated for strains CH219, K4-135, Boryong, and Ikeda using nucmer alignments filtered to retain matches with 95% nucleotide identity and 2,000 bp alignment length (**Fig 4**).

Comparisons involving strain Boryong (CH219 vs Boryong; K4-135 vs Boryong; Ikeda vs Boryong) showed highly fragmented patterns composed of multiple forward and inverted alignment blocks rather than a continuous diagonal, consistent with reduced synteny and structural differences in genome organization relative to the Ikeda-associated genomes. In contrast, the CH219 vs Ikeda comparison showed a predominant forward diagonal with limited interruptions, consistent with broadly conserved genome organization between these two genomes. Notably, the CH219 vs K4-135 and K4-135 vs Ikeda comparisons showed mixed forward and inverted alignment patterns, indicating additional structural differences despite high nucleotide similarity. Descriptive summaries of forward and reverse dot plot alignment segments are provided in **S4 Table**.

### ISfinder based identification of IS families

Using ISfinder, repeated/duplicated CDSs were further classified into major IS families (**Table 3**). Under the primary BLASTn filtering threshold (alignment length ≥ 300 bp and nucleotide identity ≥ 90%), the three Ikeda-related genomes (strains CH219, K4-135, and Ikeda) harbored comparable totals of IS-related CDSs (128–144), whereas strain Boryong contained a higher total count, consistent with its larger pool of duplicated genes. In the Ikeda-related genomes, ISOt3, ISOt4, ISOt5, and ISOt6 were more evenly represented than in strain Boryong.

**Table 3:** Copy numbers of IS families identified by ISfinder in four *O. tsutsugamushi* strains, two South Korean strains CH219 and K4-135 (this study), the Japanese reference strain Ikeda, and the South Korean reference strain Boryong.

	*O. tsutsugamushi* strains			
	strain CH219 (current study)	strain K4-135 (current study)	strain Ikeda (NC_010793.1)	strain Boryong (NC_009488.1)
Repeated genes*	1149 (126 clusters)	1199 (127 clusters)	1160 (113 clusters)	1464 (147 clusters)
ISOt3 (IS630 family)	56/35/17	34/15/6	45/28/13	3/0/0
ISOt4 (IS982 family)	30/0/0	32/0/0	33/1/0	34/0/0
ISOt5 (IS110 family)	30/30/30	32/32/32	31/31/31	13/0/0
ISOt6 (IS5 family)	28/0/0	30/0/0	29/0/0	139/73/50
Total IS elements	144/65/47	128/47/38	138/60/44	189/73/50

- IS elements were identified by BLASTN searches against the ISfinder database (E-value < 1x10^-4^), counting only the top hit per CDS.

- Values are shown as N_1_/N_2_/N_3_, where N_1_ is the number of CDSs with BLASTN hits of ≥ 300 bp alignment length and ≥ 90% nucleotide identity, N_2_ is the number with ≥ 300 bp alignment length and ≥ 99% nucleotide identity, and N_3_ is the number with ≥ 400 bp alignment length and ≥ 99% nucleotide identity.

* The numbers of repeated genes are the same as those in Table 1.

Applying increasingly stringent thresholds (≥ 300 bp and ≥ 99% identity; ≥ 400 bp and ≥ 99% identity) revealed different retention patterns across ISOt families. In the Ikeda-related genomes, ISOt5-related CDS counts remained essentially unchanged across thresholds (30–32 copies under all three criteria), indicating a highly conserved set of IS110-family elements. By contrast, most ISOt4- and ISOt6-related hits did not meet the stricter cutoffs, suggesting that many copies of these families are fragmented and/or sequence diverged. ISOt3-related CDS counts also decreased with increasing stringency but remained detectable in all three Ikeda-related genomes (17, 6, and 13 copies, respectively, at ≥ 400 bp & ≥ 99% identity).

In strain Boryong, IS family profiles responded differently to increasing stringency. ISOt3-, ISOt4-, and ISOt5-related hits dropped to zero under stricter criteria, whereas ISOt6-related hits remained the most abundant IS-related family, decreasing from 139 copies at ≥ 300 bp & ≥ 90% identity to 50 copies at ≥ 400 bp & ≥ 99% identity. Thus, even under the most stringent criteria, strain Boryong retained a relatively high number of ISOt6-related (IS5-family) hits, whereas the three Ikeda-genotype genomes retained similar numbers of ISOt5-related hits across thresholds together with variable numbers of high-identity ISOt3-related elements.

## Discussion

In this study, we generated complete WGSs of two *O. tsutsugamushi* strains, CH219 and K4-135, which are archived clinical isolates originally obtained from scrub typhus cases in South Korea in 2023 and 2024, respectively. These isolates originated from the northern region of South Korea (Gangwon and Gyeonggi provinces). Previous nationwide reports have described the Boryong strain as a predominant genotype in South Korea, while minor genotypes have been also detected, including in the northern regions [7,12]. Our findings are consistent with this pattern and indicate that minor genotypes, including the Ikeda strain, may be present in northern regions of South Korea, while the Boryong genotype remains predominant in most regions.

Genotyping based on the *56-kDa* TSA gene showed that both isolates had 100% sequence identity to the Japanese reference strain Ikeda (OTT_RS04590). The Ikeda strain has previously been detected in South Korea and associated with severe scrub typhus cases [14]. To our knowledge, this study provides one of the first complete genome analyses of clinically isolated Ikeda-related *O. tsutsugamushi* strains from South Korea. Comparative genomic analyses demonstrated that strains CH219 and K4-135 are highly similar to the Japanese strain Ikeda genome (NC_010793.1), with identical sequences at both the single-gene (*56-kDa* TSA) and MLST (seven housekeeping genes) levels. At a broader genomic scale, using recombination-filtered core-genome polymorphisms and ANI values, the two South Korean strains showed marginally greater similarity to each other than to the Japanese reference strain Ikeda, although all three strains formed a tight cluster that remained separate from the Boryong strain. This was supported by pairwise SNP distance (22 SNPs between strains CH219 and K4-135 versus 76–78 SNPs to strain Ikeda), consistent with close relatedness between the two South Korean isolates within this limited comparison. The *56kDa* TSA phylogeny in this study was constructed using a selected set of representative and geographically relevant sequences, particularly from South Korea and Japan, rather than all publicly available entries. This approach was adopted because public repositories contain a large number of *56-kDa* TSA records, many of which are partial, redundant, or uneven in sequence length and annotation quality, which would complicate direct inclusion in a single interpretable phylogenetic reconstruction.

The EggNOG-based functional categorization showed broadly similar profiles between strain CH219 and strain K4-135 indicating that these two Ikeda-related strains retain a comparable overall functional repertoire despite structural divergence. In addition to broadly similar functional profiles, in both genomes, categories S (function unknown) and L (replication, recombination and repair) were the most represented, consistent with the still limited functional annotation and highly repetitive genome architecture of *O. tsutsugamushi*.

The four compared genomes also showed substantial CDS-level redundancy, with repeated CDSs accounting for 14.5–21.7% of total genome length and 18.7–28.6% of total annotated CDS length. The highest repeated CDS burden was observed in the Boryong reference genome. These findings further support the highly repetitive genomic architecture characteristic of *O. tsutsugamushi*. Previous comparative genomic studies of *O. tsutsugamushi* have similarly reported substantial repetitive sequence burden in terms of both total length and genome proportion, including OtAGE-associated repetitive regions [16]. Although the repeat definition used here differs from those genome-wide repeat analyses, our CDS-level estimates are consistent with the general repeat-rich nature of the *O. tsutsugamushi* genome.

Because publicly available complete genomes of *O. tsutsugamushi* remain limited [20], it remains difficult to pinpoint when and where the South Korean and Japanese Ikeda lineages diverged. Nevertheless, the high overall similarity to the Japanese strain Ikeda, combined with the short but distinct branches of strains CH219 and K4-135 in the recombination-filtered core-genome phylogeny, is compatible with genome-level differentiation between the two South Korean isolates and the Japanese Ikeda reference strain, although the present dataset is too limited to infer the direction, timing, or route of spread. The circulation of *O. tsutsugamushi* may closely linked to the distribution of trombiculid mites (chiggers) and their reservoir hosts [49]. Given the geographic separation between South Korea and Japan, any transboundary spread of Ikeda-related strains would likely require occasional long-distance dispersal events. One plausible mechanism proposed in the literature is passive transport of ectoparasites via migratory birds along flyways, potentially enabling discontinuous spread between geographically separated foci [50,51]. However, our genomic data alone cannot identify the direction, timing, or route of introduction, and we therefore present this scenario only as a hypothesis consistent with the high overall similarity between the South Korean isolates and the Japanese Ikeda reference. Further evaluation would require contemporaneous sampling of vectors/reservoirs and integration with ecological and migration data, which is beyond the scope of the present study. In addition, because the analyzed isolates were archival and de-identified, we did not have access to patient travel history or other linked epidemiological metadata. Therefore, the present data cannot distinguish between recent importation and previously unrecognized long-term local circulation of Ikeda-related strains in South Korea.

Notably, dot plot comparisons showed structural differences in genome organization among the Ikeda-related genomes, with mixed forward and reverse alignment patterns between strains CH219 and K4-135 despite their close nucleotide similarity. Consistent with the highly repetitive genome architecture reported previously for *O. tsutsugamushi* [20], the two South Korean strains possessed highly repetitive genomes with large numbers of duplicated genes and multiple IS-related sequences. Using our ISfinder-based BLASTn pipeline with defined alignment length and identity thresholds, we identified differences in IS family composition between the South Korean reference strain Boryong and the Ikeda-related strains CH219 and K4-135. Among those, ISOt3- and ISOt6-related CDSs, which belong to the IS630 and IS5 families, respectively, showed the most marked contrasts. ISOt3-related CDS has been proposed to represent an ancient acquisition from chigger mites [18] and is often present in a degraded form [20], whereas ISOt6-related CDS remains highly abundant in *O. tsutsugamushi* genomes [52]. Previous studies in other bacterial systems, such as *Wolbachia*, have suggested that IS5-family elements may contribute to the mobilization of adjacent DNA segments [53]. However, these differences are best interpreted as descriptive differences in IS-related CDS composition among the compared genomes, and their structural or functional consequences were not directly assessed in this study.

Under our primary threshold (alignment length ≥ 300 bp and ≥ 90% identity), the Boryong strain harbored only three ISOt3-related copies compared with 34–56 copies in strains CH219, K4-135 and Ikeda, suggesting that most ISOt3-related CDSs are less retained or more diverged in the Boryong genome than in the Ikeda-related genomes. In contrast, ISOt6-related CDSs showed a higher observed copy number in the Boryong genome (139 copies) than in the three Ikeda-related genomes (28–30 copies) under all applied thresholds, and this enrichment of ISOt6-related CDSs in the Boryong strain remained evident under the more stringent thresholds. These observations indicate different IS-related copy-number profiles between the Boryong reference genome and the three Ikeda-related genomes in this comparison. However, no statistical or functional analysis was performed to test whether these differences reflect distinct transposition dynamics or mechanistic effects on genome rearrangement. Because our analysis relies on similarity to curated ISfinder entries and conservative filtering thresholds, the reported copy numbers should be regarded as lower-bound estimates and additional, more diverged IS-like fragments may not have been captured. Moreover, direct links between specific IS configurations and clinical or ecological phenotypes remain speculative and will require targeted functional and epidemiological studies.

More broadly, *O. tsutsugamushi* remains underrepresented in public genome databases despite its importance as an endemic pathogen causing scrub typhus throughout the Asia-Pacific region [1]. The highly repetitive nature of its genome has hampered complete genome assembly, and curated virulence factor information for *O. tsutsugamushi* is still limited. For example, it is not yet represented as a dedicated taxon in VFDB, in contrast to the related genus *Rickettsia*. Expanding the collection of complete WGS data from diverse geographic and ecological origins will enable more comprehensive comparative genomics, facilitate the identification of candidate virulence determinants and antigenic targets, and ultimately support the development of future diagnostics, vaccines, and immunological studies for scrub typhus.

This study has several limitations. First, our genomic inferences are based on only two Ikeda-related clinical isolates from the northern regions of South Korea and a single Japanese reference genome, and the currently available *O. tsutsugamushi* genomes are geographically and temporally biased. As a result, the direction and timing of divergence between South Korean and Japanese Ikeda lineages, as well as the broader population structure of Ikeda-like strains in the Asia-Pacific region, remain unresolved. Second, we did not include contemporaneous vector or reservoir isolates from chiggers or small mammals, so the proposed scenarios for dispersal and circulation could not be directly tested. Finally, we did not analyze linked clinical metadata or perform functional assays, and therefore any potential associations between specific genomic features, clinical outcome, and antigenic diversity remain speculative and will require dedicated epidemiological and experimental studies. We acknowledge that structural annotation of *O. tsutsugamushi* is intrinsically difficult because of its high repeat content and abundant pseudogenes. Although the Prokka-based workflow was suitable for standardized comparative analyses and downstream CDS-based clustering, PGAP-based annotation of the submitted genomes identified additional pseudogenes and one additional 5S rRNA locus, indicating that repetitive or degraded regions should be interpreted cautiously.

## Conclusions

In this study, we generated complete genome sequences for two *O. tsutsugamushi* isolates (strain CH219 and strain K4-135) from northern South Korea. Across *56-kDa* TSA, MLST profiles, recombination-filtered core-genome analyses, ANI, and whole-genome alignments, both isolates were closely related to the Japanese reference strain Ikeda while showing limited but detectable genome-wide divergence within this comparison. Comparison with the South Korean reference strain Boryong further revealed distinct IS-related CDS composition among the compared genomes. These findings highlight the need for expanded complete and well-supported WGS resources to better understand genome diversity and its epidemiological and biological relevance in scrub typhus.

## Supporting information

S1 Fig Circular genome maps of *O. tsutsugamushi* (a) strain CH219 and (b) strain K4-135. From the outer to inner rings, the maps show forward CDSs, reverse CDSs, tRNA genes, rRNA genes, GC content, and GC skew. Regions with GC content above the genome average are shown as outward peaks, whereas regions below the average are shown as inward peaks. Positive and negative GC skews values are shown as outward and inward peaks, respectively.
(TIF)

S2 Fig EggNOG-based functional categorization of predicted genes in *O. tsutsugamushi* (a) strain CH219 and (b) strain K4-135 with (c) EggNOG functional category defintions. The number of predicted genes assigned to each EggNOG functional category is shown for strain CH219 and strain K4-135. The two genomes exhibited broadly similar category distributions, with category S (function unknown) and category L (replication, recombination and repair) being the most represented.
(TIF)

S1 Table Basic chromosomal information of the two *O. tsutsugamushi* strains CH219 and K4-135 in South Korea and the other 16 previously reported *O. tsutsugamushi* strains. (DOCX)

S2 Table Pairwise numbers of SNP differences among CH219, K4-135, and 16 reference *Orientia tsutsugamushi* genomes calculated from the Gubbins recombination-filtered polymorphic-sites alignment. (DOCX)

S3 Table Sequence and read-mapping statistics for *O. tsutsugamushi* strains CH219 and K4-135 (Macrogen QC report). (DOCX)

S4 Table Descriptive summary statistics of pairwise whole-genome dot plot alignment segments generated by MUMmer/mummerplot. (DOCX)
