# Supplementary material for: Complete genomes of two Ikeda-genotype Orientia tsutsugamushi isolates from South Korea reveal within-lineage divergence and contrast with the Boryong reference strain
Source: PLoS One. 2026 Jul 9;21(7):e0351070. doi: 10.1371/journal.pone.0351070 (PMC13349160; doi:10.1371/journal.pone.0351070)
Supplement: S1 Table — (DOCX) [file pone.0351070.s003.docx]

| **S1 Table**. Basic chromosomal information of the two *O. tsutsugamushi* strains CH219 and K4-135 in South Korea and the other 16 previously reported *O. tsutsugamushi* strains | | | | | | | |
| --- | --- | --- | --- | --- | --- | --- | --- |
| Strain | Accession no. | Country | Host | Collection date | Length (bp) | GC ratio (%) | Assembly level |
| **strain CH219** | **Current study** | Wonju, South Korea | *Homo sapiens* | Dec 2023 | 1,978,415 | 30.5 | Complete |
| **strain K4-135** | **Current study** | Goyang, South Korea | *Homo sapiens* | Nov 2024 | 2,059,857 | 30.6 | Complete |
| strain Boryong | NC_009488.1 | Seoul, South Korea | *Homo sapiens* | 2007 | 2,127,051 | 30.5 | Complete |
| strain Ikeda | NC_010793.1 | Japan | *Homo sapiens* | 2008 | 2,008,987 | 30.5 | Complete |
| strain Wuj/2014 | NZ_CP044031.1 | Zhejiang, China | *Homo sapiens* | Jan 2014 | 1,972,387 | 30.5 | Complete |
| strain TW-1 | NZ_CP142421.1 | Lienchiang county, Taiwan | *Homo sapiens* | Aug 2007 | 2,008,429 | 30.5 | Complete |
| strain UT76 | NZ_LS398552.1 | - | - | - | 2,078,193 | 30.5 | Complete |
| strain TW-22 | NZ_CP142420.1 | Kaohsiung City, Taiwan | *Homo sapiens* | Aug 2007 | 2,044,475 | 30.5 | Complete |
| strain Kato | NZ_LS398550.1 |  |  |  | 2,319,449 | 31.0 | Complete |
| strain JJOtsu7 | NZ_CP166957.1 | Vellore, India | *Homo sapiens* | Jan 2024 | 2,183,885 | 30.5 | Complete |
| strain Karp | NZ_LS398548.1 | - | - | - | 2,469,803 | 31.0 | Complete |
| strain JJOtsu8 | NZ_CP166958.1 | Vellore, India | *Homo sapiens* | Nov 2023 | 2,344,138 | 30.5 | Complete |
| strain UT176 | NZ_LS398547.1 | - | - | - | 1,932,116 | 30.0 | Complete |
| strain JJOtsu1 | NZ_CP166954.1 | Vellore, India | *Homo sapiens* | Dec 2022 | 2,179,789 | 30.5 | Complete |
| strain JJOtsu6 | NZ_CP166956.1 | Vellore, India | *Homo sapiens* | Nov 2023 | 2,284,757 | 30.5 | Complete |
| strain Gilliam | NZ_LS398551.1 | - | - | - | 2,465,012 | 30.5 | Complete |
| strain JJOtsu5 | NZ_CP166955.1 | Vellore, India | *Homo sapiens* | Nov 2023 | 2,446,845 | 30.5 | Complete |
| strain TA686 | NZ_LS398549.1 | - | - | - | 2,254,553 | 30.5 | Complete |
| Metadata in this table were compiled form the corresponding public WGS-associated records. Empty entries indicate information not available in those records at the time of data retrieval. Information from unrelated strain reports or non-WGS studies was not used to supplement missing metadata. | | | | | | | |
