# Supplementary material for: Complete genomes of two Ikeda-genotype Orientia tsutsugamushi isolates from South Korea reveal within-lineage divergence and contrast with the Boryong reference strain
Source: PLoS One. 2026 Jul 9;21(7):e0351070. doi: 10.1371/journal.pone.0351070 (PMC13349160; doi:10.1371/journal.pone.0351070)
Supplement: S2 Table — (DOCX) [file pone.0351070.s004.docx]

| **S2 Table**. Pairwise numbers of SNP differences among CH219, K4-135, and 16 reference *Orientia tsutsugamushi* genomes calculated from the Gubbins recombination-filtered polymorphic-sites alignment. | | | | | | | | | | | | | | | | | | |
| --- | --- | --- | --- | --- | --- | --- | --- | --- | --- | --- | --- | --- | --- | --- | --- | --- | --- | --- |
| Strains | strain_CH219 | strain_K4-135 | strain_Ikeda | strain_Boryong | strain_TA686 | strain_Gilliam | strain_JJOtsu1 | strain_JJOtsu5 | strain_JJOtsu7 | strain_JJOtsu8 | strain_JJOtsu6 | strain_Karp | strain_Kato | strain_TW-1 | strain_TW-22 | strain_UT76 | strain_UT176 | strain_Wuj_2014 |
| strain_CH219 | 0 | 22 | 78 | 10598 | 8076 | 7201 | 7824 | 7778 | 6004 | 6259 | 7829 | 6585 | 2429 | 4976 | 5751 | 6084 | 7070 | 4978 |
| strain_K4-135 | 22 | 0 | 76 | 10491 | 7971 | 7183 | 7745 | 7737 | 5980 | 6200 | 7750 | 6508 | 2394 | 4951 | 5707 | 6035 | 7004 | 4949 |
| strain_Ikeda | 78 | 76 | 0 | 10630 | 8115 | 7321 | 7813 | 7804 | 6029 | 6211 | 7818 | 6512 | 2449 | 5013 | 5748 | 6109 | 7057 | 5018 |
| strain_Boryong | 10598 | 10491 | 10630 | 0 | 13930 | 12876 | 13307 | 13345 | 12579 | 12391 | 13310 | 12759 | 11721 | 10634 | 12374 | 12004 | 12742 | 10622 |
| strain_TA686 | 8076 | 7971 | 8115 | 13930 | 0 | 10774 | 11072 | 11197 | 9436 | 9362 | 11077 | 9752 | 8883 | 7737 | 9121 | 8685 | 9999 | 7780 |
| strain_Gilliam | 7201 | 7183 | 7321 | 12876 | 10774 | 0 | 10360 | 10459 | 8942 | 8888 | 10362 | 9383 | 8003 | 7586 | 8823 | 8821 | 9739 | 7548 |
| strain_JJOtsu1 | 7824 | 7745 | 7813 | 13307 | 11072 | 10360 | 0 | 8382 | 8995 | 8773 | 3 | 9569 | 8590 | 7872 | 9303 | 9029 | 9182 | 7819 |
| strain_JJOtsu5 | 7778 | 7737 | 7804 | 13345 | 11197 | 10459 | 8382 | 0 | 9099 | 9020 | 8386 | 9690 | 8685 | 7992 | 9345 | 9182 | 9274 | 7951 |
| strain_JJOtsu7 | 6004 | 5980 | 6029 | 12579 | 9436 | 8942 | 8995 | 9099 | 0 | 6623 | 8998 | 8004 | 6862 | 6120 | 7254 | 7164 | 8328 | 6154 |
| strain_JJOtsu8 | 6259 | 6200 | 6211 | 12391 | 9362 | 8888 | 8773 | 9020 | 6623 | 0 | 8776 | 7802 | 6890 | 5861 | 7379 | 6856 | 8221 | 5859 |
| strain_JJOtsu6 | 7829 | 7750 | 7818 | 13310 | 11077 | 10362 | 3 | 8386 | 8998 | 8776 | 0 | 9573 | 8594 | 7875 | 9307 | 9035 | 9186 | 7822 |
| strain_Karp | 6585 | 6508 | 6512 | 12759 | 9752 | 9383 | 9569 | 9690 | 8004 | 7802 | 9573 | 0 | 7256 | 6449 | 7608 | 7510 | 8381 | 6426 |
| strain_Kato | 2429 | 2394 | 2449 | 11721 | 8883 | 8003 | 8590 | 8685 | 6862 | 6890 | 8594 | 7256 | 0 | 5575 | 6444 | 6644 | 7790 | 5586 |
| strain_TW-1 | 4976 | 4951 | 5013 | 10634 | 7737 | 7586 | 7872 | 7992 | 6120 | 5861 | 7875 | 6449 | 5575 | 0 | 5465 | 3737 | 6605 | 242 |
| strain_TW-22 | 5751 | 5707 | 5748 | 12374 | 9121 | 8823 | 9303 | 9345 | 7254 | 7379 | 9307 | 7608 | 6444 | 5465 | 0 | 6742 | 8132 | 5530 |
| strain_UT76 | 6084 | 6035 | 6109 | 12004 | 8685 | 8821 | 9029 | 9182 | 7164 | 6856 | 9035 | 7510 | 6644 | 3737 | 6742 | 0 | 7411 | 3821 |
| strain_UT176 | 7070 | 7004 | 7057 | 12742 | 9999 | 9739 | 9182 | 9274 | 8328 | 8221 | 9186 | 8381 | 7790 | 6605 | 8132 | 7411 | 0 | 6661 |
