# Supplementary material for: Complete genomes of two Ikeda-genotype Orientia tsutsugamushi isolates from South Korea reveal within-lineage divergence and contrast with the Boryong reference strain
Source: PLoS One. 2026 Jul 9;21(7):e0351070. doi: 10.1371/journal.pone.0351070 (PMC13349160; doi:10.1371/journal.pone.0351070)
Supplement: S3 Table — (DOCX) [file pone.0351070.s005.docx]

| **S3 Table**. Sequence and read-mapping statistics for *O. tsutsugamushi* strains CH219 and K4-135 (Macrogen QC report). | | | |
| --- | --- | --- | --- |
| Metric | Strain | | |
|  | CH219 | K4-135 |  |
| PacBio HiFi reads | 110,128 | 76,438 |  |
| PacBio total bases | 956.9 Mb | 694.1 Mb |  |
| PacBio read N50 | 10,281 bp | 10,724 bp |  |
| PacBio mapped reads | 35,395 (32.1%) | 34,508 (45.1%) |  |
| PacBio average depth | 169.6x | 164.8x |  |
| PacBio coverage | 100% | 100% |  |
| Illumina read pairs | 19,175,094 | 16,957,842 |  |
| Illumina total bases (after QC) | 2.8 Gb | 2.5 Gb |  |
| Illumina mapped reads | 5,355,442 (27.9%) | 6,899,490 (40.6%) |  |
| Illumina average depth (after QC) | 393.9x | 475.5x |  |
| Illumina coverage | 100% | 100% |  |
| Hifiasm contigs^a^ | 1 | 1 |  |
| Contig N50^b^ | 1,978,415 bp | 2,059,857 bp |  |
| Final chromosome length | 1,978,415 bp | 2,059,857 bp |  |
| Mb, megabases; Gb, gigabases, bp, base pairs, x, fold coverage.  ^a^, Assemblies consisted of a single contig per strain  ^b^, Contig N50 is therefore equal to the final chromosome length | | | |
