# Supplementary material for: Complete genomes of two Ikeda-genotype Orientia tsutsugamushi isolates from South Korea reveal within-lineage divergence and contrast with the Boryong reference strain
Source: PLoS One. 2026 Jul 9;21(7):e0351070. doi: 10.1371/journal.pone.0351070 (PMC13349160; doi:10.1371/journal.pone.0351070)
Supplement: S4 Table — (DOCX) [file pone.0351070.s006.docx]

| **S4 Table**. Descriptive summary statistics of pairwise whole-genome dot plot alignment segments generated by MUMmer/mummerplot. | | | | | | | |
| --- | --- | --- | --- | --- | --- | --- | --- |
| Reference strain | Query strain | No. of forward segments | Total forward segment length (bp) | Maximum forward segment length (bp) | No. of reverse segments | Total reverse segment length (bp) | Maximum reverse segment length (bp) |
| Boryong | CH219 | 43 | 311,635 | 21,140 | 45 | 323,955 | 25,169 |
| Ikeda | CH219 | 52 | 1,582,293 | 94,253 | 14 | 347,296 | 87,435 |
| Boryong | K4-135 | 51 | 352,834 | 26,140 | 41 | 287,813 | 21,324 |
| Ikeda | K4-135 | 25 | 1,054,294 | 136,195 | 33 | 957,903 | 103,365 |
| Ikeda | Boryong | 40 | 287,428 | 26,463 | 51 | 353,405 | 25,181 |
| CH219 | K4-135 | 18 | 673,431 | 184,623 | 35 | 1,338,022 | 179,623 |
| Forward and reverse segments were summarized from mummerplot output files (.fplot and .rplot) generated from MUMmer alignments. Segment lengths represent plot-derived alignment spans and were used as descriptive summaries of dot plot structure; they do not represent formal breakpoint-define inversion sizes or validated structural variant lengths. | | | | | | | |
